# Dietary linseed oil affects the polyunsaturated fatty acid and transcriptome profiles in the livers and breast muscles of ducks

**DOI:** 10.3389/fnut.2022.1030712

**Published:** 2022-10-28

**Authors:** Laidi Wang, Bingqiang Dong, Ting Yang, Ao Zhang, Xiaodan Hu, Zhixiu Wang, Guobin Chang, Guohong Chen

**Affiliations:** Key Laboratory of Animal Genetics and Breeding and Molecular Design of Jiangsu Province, Yangzhou University, Yangzhou, China

**Keywords:** n-3 PUFA, n-6 PUFA, *FADS*, *CD36*, duck

## Abstract

Linseed oil, an important source of dietary α-linolenic acid, is used to provide meat enriched in n-3 PUFA. We investigated the effects of dietary linseed oil (0, 0.5, 1, and 2%) on growth performance, meat quality, tissue fatty acid (FA), and transcriptome profiles in ducks. The result showed that dietary linseed oil had no effect on growth performance. Increasing dietary linseed oil enrichment raised n-3 PUFA and linoleic acid (LA) levels in both the liver and breast muscle, but decreased dihomo-gamma-linolenic acid (DGLA) and arachidonic acid (ARA) levels in the liver. The liver n-3 PUFA content was negatively correlated with duck body weight. Transcriptome analysis showed that dietary linseed oil caused hepatic changes in genes (*SCD*, *FADS1*, *FADS2*, and *ACOT6*) related to the biosynthesis of unsaturated fatty acids. Besides, dietary linseed oil also affected the expression of genes related to PUFAs and downstream metabolites (such as linoleic acid, steroid hormone, progesterone, etc.) metabolic pathways in both liver and breast muscle. Key genes involved in PUFA synthesis and transport pathways were examined by RT-qPCR, and the results verified that hepatic expression levels of *FADS1* and F*ADS2* decreased, and those of *FABP4* and *FABP5* increased when 2% linseed oil was added. *CD36* expression level increased in breast muscle when 2% linseed oil was added. Thus, 2% dietary linseed oil supplementation produces n-3 PUFA-enriched duck products by regulating the PUFA metabolic pathways, which could be advantageous for health-conscious consumers.

## Introduction

Polyunsaturated fatty acids (PUFAs), classified into n-3 and n-6 PUFAs, influence growth, development, reproduction, and vulnerability to diseases. Linoleic acid (LA), dihomo-gamma-linolenic acid (DGLA), and arachidonic acid (ARA) are n-6 PUFAs, whereas α-linolenic acid (ALA), eicosapentaenoic acid (EPA), and docosahexaenoic acid (DHA) belong to n-3 PUFAs (1). Both types of fatty acids (FAs) are precursors of signaling molecules with opposing effects that regulate membrane microdomain composition, receptor signaling, and gene expression (2). LA and ALA are derived from lipids of vegetable origin and are considered essential because they cannot be synthesized *de novo* in vertebrates. The conversion of the n-6 fatty acid LA to ARA plays a role in inducing inflammation, adipogenesis, and regulation of the endocannabinoid system (3). n-3 PUFAs decrease inflammatory signaling and lipid synthesis and stimulate fatty acid degradation (4). In humans, the optimal n-6:n-3 PUFA ratio is 5–10, whereas in popular modern western diets, the ratio is 15–16.7 (5). Excessive intake of n-6 PUFA and a very high n-6/n-3 PUFA ratio in the food increase the risk of obesity, cardiovascular diseases, and cancer (3, 6). It is necessary to improve human nutrition by supplying n-3 PUFA enriched foods.

Duck meat is popular in both Asian and European countries. Compared to chicken meat, ducks have a higher content of red muscle fibers in breast muscles (7). While the n-6: n-3 PUFA ratio in duck is about 15–30 under a regular diet pattern, which does not meet human optimal health requirements. ALA, the plant omega-3, is enriched in flaxseed, perilla, echium, walnut, and algal oil (8), and fish oils that contain high contents of EPA and DHA are typically the most abundantly available (9). Therefore, it is important to consider the quality of added fats in duck diet, and duck meat could be a viable source of n-3 PUFAs for humans. On the other hand, ducks have a high ability to efficiently convert dietary ALA to DHA (10). The conversion occurs through fatty acid desaturases (encoded by *FADS1* and *FADS2*) and elongases (encoded by *ELOVL5* and *ELOVL2*) in the liver (11) and is subsequently supplied (*via* blood circulation) to other tissues (12). Long-chain fatty acid (LCFA) uptake occurs not only through free diffusion across the plasma membrane, but also by a protein-mediated mechanism (13). Their translocation depends on fatty acid transporters, such as fatty acid transport proteins/solute carrier 27A (FATP/SLC27A), fatty acid translocase/cluster of differentiation 36 (FAT/CD36), caveolin-1 (CAV1), and fatty acid binding protein (FABP) (13). Although PUFA composition in ducks has been studied, the mechanism of PUFA synthesis and transport in ducks has not been fully elucidated.

Recently, linseed oil has been incorporated into foods to improve the cardiovascular health of humans (14). Considering the high cost of fish oil, linseed oil is also an ideal source of n-3 PUFA in animal diets. In poultry, linseed oil or flaxseed-supplemented diets increase n-3 PUFA levels and decrease n-6:n-3 PUFA ratios in both eggs and meat, providing products enriched in n-3 PUFA, for which many positive health effects have been described (15–17). However, little attention has been paid to the combined effect of diets formulated with linseed oil on gene transcription in ducks, as well as to their effects on polyunsaturated fatty acid metabolism in ducks. Therefore, in this study, Runzhou white-crested ducks, a popular duck breed with a small body size, were used to assess the effect of a linseed-oil-enriched diet on the performance, meat quality, genes transcriptome and lipid metabolism. These insights might provide feasible method in producing n-3 PUFA enriched ducks, and key genes involved in PUFA synthesis and transport pathways were evaluated in linseed oil-administered ducks.

## Materials and methods

### Experimental design and diets

A total of 224 28-day old Runzhou white-crested ducks of the commodity generation (average weight: 753.35 ± 12.94 g) were randomly divided into four treatment groups: control (basal diet + 2% duck oil), low linseed oil (basal diet + 1.5% duck oil + 0.5% linseed oil), medium linseed oil (basal diet + 1% duck oil + 1% linseed oil), and high linseed oil (basal diet + 2% linseed oil). Each treatment group had four replicates, each consisting of 14 ducks (half male and half female). The ducks from one batch were fed the same diet at 1–28 days of age. The four treatment diets were formulated by adding different proportions of duck oil and linseed oil (Table 1). Duck oil was gradually replaced with linseed oil to achieve a higher ALA content and lower n-6/n-3 PUFA ratio. The fatty acid composition of the experimental diets and oils are presented in Table 2. The ALA content in linseed oil was 35.21%, and with an increase in linseed oil, the ALA level increased gradually from 0.0766 to 0.6816 g/100 g, and the n-6/n-3 PUFA ratio decreased gradually from 17.37 to 1.97, as expected. The birds were raised in deep litter comprising sawdust and paddy husks. All ducks had *ad libitum* access to food and water. Considering the previous research (17) and the characteristics of small size meat ducks, the feeding trial lasted for 28 days. None of the ducks died during the 28 days experiment. All ducks were provided by Zhenjiang Tiancheng Agricultural Technology, Jiangsu Province, where the experiments were also conducted.

**TABLE 1:** Composition and nutrient level of diets.

Item	Control^2^	High^2^	Medium^2^	Low^2^
**Ingredient (%)**				
Corn	47.16	47.16	47.16	47.16
Soybean meal	24.23	24.23	24.23	24.23
Flour	17.66	17.66	17.66	17.66
Rice bran	5.00	5.00	5.00	5.00
Duck oil	2.00	1.50	1.00	0.00
Linseed oil	0.00	0.50	1.00	2.00
Dicalcium phosphate	1.65	1.65	1.65	1.65
Limestone	1.00	1.00	1.00	1.00
Salt	0.30	0.30	0.30	0.30
Premix^1^	1.00	1.00	1.00	1.00
Total	100	100	100	100
**Nutrient level**				
AME (MJ/kg)	11.31	11.31	11.31	11.31
CP (%)	17.52	17.52	17.52	17.52
EE (%)	5.76	5.77	5.77	5.77
CF (%)	3.59	3.59	3.59	3.59
Ca (%)	0.89	0.89	0.89	0.89
Total phosphorus (%)	0.75	0.75	0.75	0.75
Lys (%)	0.90	0.90	0.90	0.90

^1^Vitamin and mineral premixes supplied per kilogram diet: vitamin A, 9,000 IU; vitamin E, 79 mg; vitamin D_3_, 3,000 IU; vitamin B_2_, 8 mg; vitamin K_3_, 2 mg; pantothenic acid, 3.2 mg; niacin, 11 mg; biotin, 1 mg; folic acid, 1.5 mg; Zn, 60 mg; Mn, 49 mg; Co, 1 mg; Cu, 6 mg; I, 2 mg; Se, 0.18 mg. ^2^Control: control group (basal diet + 2% duck oil), Low: low linseed oil group (basal diet + 1.5% duck oil + 0.5% linseed oil), Medium: medium linseed oil group (basal diet + 1% duck oil + 1% linseed oil), and High: high linseed oil group (basal diet + 2% linseed oil), as described below.

**TABLE 2:** Fatty acid (FA) composition of diets.

Fatty acids composition	Content (g/100 g)					
	
	Control	High	Medium	Low	Duck oil	Linseed oil
TFA	5.00	4.94	4.72	4.87	70.32	68.13
SFA	1.55	1.48	1.37	1.29	19.94	7.17
MUFA	1.91	1.81	1.65	1.53	32.70	13.82
PUFA	1.5420	1.6526	1.7002	2.0494	17.6904	47.1457
LA (C18:2n6c)	1.4324	1.4098	1.3149	1.3398	16.3982	11.2419
GLA (C18:3n6)	ND	ND	ND	ND	0.0389	0.4143
DGLA (C20:3n6)	0.0060	0.0089	0.0074	0.0072	0.0770	0.0462
ARA (C20:4n6)	0.0091	0.0092	0.0075	0.0058	0.2417	0.0444
Total n-6 PUFA	1.4476	1.4234	1.3261	1.3527	16.7558	11.7468
ALA (C18:3n3)	0.0766	0.2126	0.3573	0.6816	0.6694	35.2069
EPA (C20:5n3)	ND	ND	ND	ND	0.0453	0.0468
DHA (C22:6n3)	0.0067	0.0063	0.0056	0.0058	0.1001	0.0883
Total n-3 PUFA	0.0833	0.2188	0.3645	0.6874	0.8227	35.3749
n-6/n-3 PUFA	17.37	6.51	3.64	1.97	20.37	0.33

g/100 g, denominator represents fresh sample weight; ND means below the detection limit, not detected, because PUFA content is low and four decimal places are reserved; TFA, total fatty acids; SFA, saturated fatty acid; MUFA, monounsaturated fatty acids; PUFA, polyunsaturated fatty acids; n-6/n-3 PUFA, ratio of n-6 PUFA and n-3 PUFA; LA, linoleic acid; GLA, gamma linolenic acid; DGLA, dihomo-gamma-linolenic acid; ARA, arachidonic acid; ALA, α-linolenic acid; EPA, eicosapentaenoic acid; DHA, docosahexaenoic acid, as described below.

### Production performance and sample collection

Following a 12-h fast, the duck body weight (BW), and feed intake (28–56 days of age) were recorded, and average daily feed intake (ADFI), average daily weight gain (ADG), and average feed/gain (F/G) were calculated. Six ducks from each replicate (three male and three female) were randomly selected and killed by stunning at the end of the experiment. Four small portions of the right breast muscle and right liver were collected, immediately placed in liquid N_2_, and stored at –80°C for fatty acid and gene expression analyses. A large portion of the left breast muscle was collected and stored at –80°C.

### Meat quality indexes

At the time of debonding (24 h post-mortem), the whole left breast muscle was used to determine physical properties (meat color, pH values, water loss rate, and shearing force values) analysis of meat. The rest of the left breast muscle was frozen at –20°C.

Meat color was measured by the optical reflection method using the CIELAB system (CIE, 1978) with L* (lightness), a* (redness), and b* (yellowness) measured using a colorimeter (CR-400, Konica Minolta, Japan). Color measurements were taken on the medial surface of each left breast or thigh and averaged. The pH of the upper, middle, and lower portions of the left breast muscles was determined in triplicate using a glass electronic pH meter (DELTA 320; Mettler Toledo, Switzerland) inserted 10 mm into the breast muscles.

After cooking in a water bath (20 min at 80°C), the breast filets were cooled down, and at least three cubes of approximately 30 g were cut from the breast filets. The water-holding capacity was determined using a hydraulic machine (M10, Bulader, Beijing, China) equipped with a 35 kg load for 5 min. Water loss rate was calculated by one minus the water-holding capacity. The water-holding capacity. At least three cylinders (3 cm in length, 1 cm in width, and 1 cm in height) were cut from the samples. The cylinders were then sheared perpendicular to the longitudinal route of the fibers loaded with a maximum 500-N load cell and a crosshead speed of 200 mm/min using a tenderness meter (C-LM3, Tenovo, Beijing, China). Subsequently, the shear forces of the cylinders were recorded. A FoodScan Meat Product Analyzer (Foss, Denmark) was used for chemical composition detection (18). After the removing of membranes, tendons, and visible fat were removed, the meat was homogenized and prepared for moisture, protein, intramuscular fat (IMF), and collagen analyses. Measurements were taken using a mixture of three individual ducks from the same replicate group, and the analysis was repeated three times.

### Fatty acid analysis

For FA analysis, approximately 100 mg of feed, liver, or breast muscle was weighed and hydrolyzed, extracted, saponified, and methylated. The FA methyl esters were subjected to Trace1310-ISQ gas chromatography and mass spectrometry (Thermos, Waltham, MA, USA) using an HP-88 column (100 m × 0.25 mm × 0.20 μm, Agilent, Santa Clara, CA, USA) for fatty acid separation. The column oven temperature was maintained at 100°C for 15 min after sample injection and was programmed to increase from 100 to 190°C at 15°C/min and maintained at 190°C for 25 min. Then the temperature was increased to 235°C at a rate of 2.5°C/min and held for 4 min. The injector and detector temperatures were 240 and 280°C, respectively. Helium was used as the carrier gas at a flow rate of 1.0 ml/min. Fatty acids were identified and quantified by comparing the retention times and peak areas with those of standard components (Sigma, Louis, MO, USA). The results were recorded as a percentage of fresh feed or breast muscle.

### Transcriptome analysis

Total RNA of liver and breast muscle samples was extracted by a Trizol reagent (Invitrogen, Carlsbad, CA, USA) according to the manufacturer’s protocol. mRNA was purified from total RNA using poly-T oligo-attached magnetic beads. And then the mRNA was randomly segmented into small fragments by divalent cations in a fragmentation buffer. First strand cDNA was synthesized using random hexamer primer. Second strand cDNA synthesis was subsequently performed using DNA Polymerase I and RNase H. The cDNA fragments were purified with AMPure XP system (Beckman Coulter, Beverly, MA, USA), and then connected with sequencing adapters according to an Illumina protocol (San Diego, CA, USA). After quality assessment, the target fragments of 250–300 bp were selected for PCR amplification to create the final cDNA library. The sequencing and analysis were performed by the Novogene Bioinformatics Technology Co., Ltd.

Clean reads were obtained by removing reads containing adapter, reads containing ploy-N and low quality reads from raw data. All samples had a Q20 (bases of Q ≥ 20/all bases of sequencing) of >96%. Clean data were mapped to the duck reference genome^1^ by Hisat2 (version: 2.0.5). mRNA levels was quantified by the value of fragments per kilobase of exon per million mapped reads (FPKM). Differential expression analysis of the high linseed oil group vs. control group was performed using the DESeq2 R package (version 1.24.0) (19). Genes with FDR (adjusted *p*-value) less than 0.05 and absolute log2-fold change more than 1 were assigned as DEGs.

Gene Ontology (GO) enrichment analysis of differentially expressed genes was implemented by the clusterProfiler R package. GO terms with *P* < 0.05 were considered significantly enriched by differential expressed genes. Kyoto Encyclopedia of Genes and Genomes (KEGG)^2^ is a database resource for pathway analysis. ClusterProfiler R package is used to test the statistical enrichment of differential expression genes in KEGG pathways.

### Gene expression validation using RT-qPCR

Total RNA was extracted from breast muscle using TRIzol reagent (Takara, Osaka, Janpan). Extracted RNA was quantified and reverse-transcribed into cDNA using PrimeScript RT-PCR Kit (TaKaRa, Osaka, Japan). mRNA expression of genes was assessed using the QuantStudio 5 Real-Time PCR detection system (Applied Biosystems, Foster, CA, USA) in a final volume of 20 μl using the SYBR Premix Ex Taq II kit (Takara, Osaka, Japan). The relative mRNA abundance of nine PUFA-synthesis-related genes was assayed. The primers used are listed in Supplementary Table 1. Glyceraldehyde-3-phosphatedehydrogenase (*GAPDH*) and Beta-actin (β*-ACTIN*) were used as internal controls, and all assays were performed in triplicates. Relative transcriptional alterations were evaluated using 2^–ΔΔ*Ct*^.

### Statistical analysis

The data were analyzed by one-way ANOVA using SPSS (version 17.0) in a completely randomized design with a model containing treatment as the main effect. The pen was used as the experimental unit to measure live weight, feed intake, and FCR. For the other indices, individual ducks were considered as experimental units. Data are expressed as mean ± standard deviation. Differences among the groups were considered significant at *P* < 0.05. When the treatment effect was significant, differences among the treatment means were detected using Duncan’s test.

## Results

### Growth performance and meat quality

The data presented in Table 3 show that dietary linseed oil had no effect on the BW of ducks at 56 days of age. Additionally, dietary linseed oil had no effect on the ADFI, ADG, or F/G of ducks at 28–56 days of age (*P* > 0.05). Physical properties (color, pH, water loss rate, and shear force) and chemical composition (moisture, protein, IMF, and collagen contents) of breast muscle in the linseed oil-supplemented diet groups are shown in Table 4. There were also no significant differences in these breast muscle indexes among groups fed different percentages of linseed oil diets (*P* > 0.05).

**TABLE 3:** Effects of dietary linseed oil supplementation on growth performance of 28–56 days ducks.

Item	Control	Low	Medium	High	*P*-value
BW (kg)	1.49 ± 0.05	1.45 ± 0.04	1.44 ± 0.03	1.42 ± 0.01	0.128
ADFI (g/d)	98.21 ± 4.05	95.92 ± 1.22	96.61 ± 1.44	96.26 ± 3.47	0.734
BWG (g/d)	26.19 ± 1.58	25.08 ± 1.28	24.28 ± 0.78	24.04 ± 0.26	0.102
F/G	3.75 ± 0.08	3.83 ± 0.17	3.98 ± 0.08	4.01 ± 0.15	0.093

Values are expressed as means ± standard deviation, *n* = 4. Mean values marked with different letters in the same line indicate significant differences, *P* < 0.05.

**TABLE 4:** Effects of different dietary linseed oil levels on breast muscle quality of 56 days ducks.

Item		Control	Low	Medium	High	*P*-value
Physical properties	L*	40.20 ± 2.86	40.63 ± 2.81	39.43 ± 2.94	45.31 ± 3.79	0.088
	a*	16.87 ± 1.42	17.22 ± 2.67	16.73 ± 1.90	16.60 ± 0.28	0.975
	b*	6.02 ± 1.40	5.87 ± 0.48	5.63 ± 2.25	6.54 ± 2.10	0.938
	pH_24*h*_	6.00 ± 0.07	6.17 ± 0.09	6.08 ± 0.11	6.10 ± 0.10	0.074
	Water loss rate (%)	23.85 ± 7.69	23.04 ± 5.20	25.89 ± 2.30	25.52 ± 3.48	0.908
	Shear force (N)	26.59 ± 9.30	23.57 ± 4.84	31.82 ± 2.38	25.30 ± 5.95	0.514
Chemical composition	Moisture (%)	75.57 ± 0.57	74.15 ± 1.87	75.30 ± 0.29	75.17 ± 0.69	0.189
	Protein (%)	22.16 ± 0.88	23.58 ± 0.60	23.42 ± 0.46	22.90 ± 0.42	0.406
	IMF (%)	1.82 ± 0.19	1.94 ± 0.30	2.02 ± 0.25	1.79 ± 0.07	0.256
	Collagen (%)	1.07 ± 0.37	1.40 ± 0.33	1.03 ± 0.30	1.05 ± 0.41	0.652

Values are expressed as means ± standard deviation, *n* = 24 for physical properties analysis, *n* = 8 for chemical composition. Mean values marked with different letters in the same line indicate significant differences, *P* < 0.05. L* = Lightness, a* = Redness, b* = Yellowness.

### Fatty acid profile in the liver

The effects of dietary linseed oil on the FA composition in the liver are presented in Table 5. Different levels of dietary linseed oil had no effect (*P* > 0.05) on total saturated FA (SFA) or monounsaturated fatty acid (MUFA) concentrations in the liver. With the increase in dietary linseed oil supplement, the concentration of PUFA tended (*P* < 0.001) to increase significantly, and the contents of total n-3 PUFA, ALA, EPA, and DHA were significantly higher (*P* < 0.001) in the low, medium, and high groups than in the control group. There were no significant differences (*P* > 0.05) in total n-6 PUFA content among the groups. However, the DGLA and ARA contents were significantly lower (*P* < 0.01) in the medium and high groups than in the control group, whereas the LA content was significantly higher (*P* < 0.001) in the medium and high groups than in the low and control groups. Therefore, in the liver, the n-6 to n-3 PUFA ratio increased (*P* < 0.001) by 13.98, 8.70, 6.55, and 4.38 for ducks fed the control, low, medium, and high linseed oil diets, respectively.

**TABLE 5:** Effects of different linseed oil levels on the polyunsaturated fatty acid (PUFA) profile of duck liver.

FA composition (mg/100 g)	Control	High	Medium	Low	*P*-value
TFA	238.11 ± 22.74^b^	261.13 ± 43.03^ab^	277.63 ± 52.20^a^	291.54 ± 72.72^a^	0.011
SFA	102.43 ± 9.56	107.53 ± 14.11	115.48 ± 19.99	116.49 ± 28.63	0.100
MUFA	40.33 ± 11.44	39.14 ± 11.82	49.44 ± 18.03	49.62 ± 23.03	0.060
PUFA	99.68 ± 4.66^c^	106.99 ± 11.25^bc^	112.69 ± 16.07^b^	125.47 ± 23.63^a^	<0.001
LA (C18:2n6c)	26.65 ± 5.26^b^	28.63 ± 5.61^b^	37.63 ± 10.57^a^	43.23 ± 14.47^a^	<0.001
DGLA (C20:3n6)	1.94 ± 0.76^a^	1.63 ± 0.38^b^	1.44 ± 0.37^b^	1.50 ± 0.46^b^	0.005
ARA (C20:4n6)	64.57 ± 8.41^a^	66.78 ± 8.32^a^	58.13 ± 7.15^b^	56.62 ± 7.48^b^	<0.001
Total n-6 PUFA	92.54 ± 3.33	96.03 ± 10.93	97.31 ± 14.20	100.38 ± 19.77	0.399
ALA (C18:3n3)	0.87 ± 0.31^d^	1.62 ± 0.65^c^	3.33 ± 1.47^b^	8.32 ± 3.76^a^	<0.001
EPA (C20:5n3)	0.49 ± 0.17^c^	0.77 ± 0.22^b^	0.84 ± 0.29^b^	1.89 ± 0.53^a^	<0.001
DHA (C22:6n3)	5.50 ± 1.56^d^	8.56 ± 2.60^c^	10.11 ± 2.25^b^	13.57 ± 3.40^a^	<0.001
Total n-3 PUFA	6.86 ± 1.81^d^	11.18 ± 2.64^c^	14.28 ± 3.19^b^	23.84 ± 6.24^a^	<0.001
n-6/n-3 PUFA	13.98 ± 2.8^a^	8.70 ± 1.41^b^	6.55 ± 0.83^c^	4.38 ± 1.03^d^	<0.001

The denominator 100 g of mg/100 g refers to the fresh sample weight, and the value is expressed as mean ± standard deviation, *n* = 24. Mean values marked with different letters in the same line indicate significant differences (*P* < 0.05). TFA, total fatty acids; SFA, saturated fatty acid; MUFA, monounsaturated fatty acids; PUFA, polyunsaturated fatty acids; n-6/n-3 PUFA, ratio of n-6 PUFA and n-3 PUFA; LA, linoleic acid; GLA, gamma linolenic acid; DGLA, dihomo-gamma-linolenic acid; ARA, arachidonic acid; ALA, a-linolenic acid; EPA, eicosapentaenoic acid; DHA, docosahexaenoic acid, as described below.

### Fatty acid profile in the breast muscle

In the breast muscle, different levels of linseed oil diets had no effect (*P* > 0.05) on the concentrations of TFA, SFA, MUFA, n-6 PUFA, or ARA (Table 6). However, the contents of PUFA, LA, and DGLA increased (*P* < 0.05) with the increase in dietary linseed oil level. The concentrations of n-3 PUFA, ALA, and DHA were significantly (*P* < 0.001) higher in the control vs. low, low vs. medium, and medium vs. high dietary linseed oil. EPA was not detected in breast muscle in the control or low linseed oil groups, and it increased significantly (*P* < 0.001) in the medium vs. high linseed oil groups. In contrast, the proportion of n-6/n-3 PUFA ratios in the breast muscle decreased (*P* < 0.001) from 32.94 to 8.54 with increasing dietary linseed oil ratios.

**TABLE 6:** Effects of different dietary linseed oil levels on the polyunsaturated fatty acid (PUFA) profile of duck breast muscle.

Fatty acid composition (mg/100 g)	Control	High	Medium	Low	*P*-value
TFA	72.26 ± 10.16	75.43 ± 13.89	79.71 ± 15.26	80.94 ± 17.83	0.175
SFA	28.97 ± 3.79	30.61 ± 5.10	32.22 ± 6.30	31.46 ± 7.80	0.301
MUFA	11.66 ± 2.93	10.93 ± 3.36	11.42 ± 2.29	12.14 ± 2.66	0.560
PUFA	31.63 ± 5.15^b^	33.89 ± 6.88^ab^	35.65 ± 7.03^ab^	37.34 ± 8.33^a^	0.043
LA (C18:2n6c)	13.00 ± 2.33^b^	15.39 ± 4.24^a^	15.61 ± 3.83^a^	16.06 ± 4.20^a^	0.029
DGLA (C20:3n6)	0.49 ± 0.17^a^	0.38 ± 0.11^b^	0.50 ± 0.16^a^	0.54 ± 0.11^a^	0.004
ARA (C20:4n6)	16.79 ± 2.91	16.16 ± 2.49	17.13 ± 2.51	16.38 ± 3.62	0.686
Total n-6 PUFA	30.20 ± 4.65	31.90 ± 6.42	32.91 ± 6.10	32.94 ± 7.47	0.416
ALA (C18:3n3)	0.12 ± 0.20^d^	0.38 ± 0.31^c^	0.60 ± 0.22^b^	1.18 ± 0.50^a^	<0.001
EPA (C20:5n3)	ND	ND	0.20 ± 0.22^b^	0.60 ± 0.16^a^	<0.001
DHA (C22:6n3)	0.89 ± 0.26^d^	1.29 ± 0.23^c^	1.80 ± 0.49^b^	2.32 ± 0.54^a^	<0.001
Total n-3 PUFA	1.01 ± 0.4^a^	1.68 ± 0.44^b^	2.55 ± 0.87^c^	4.00 ± 1.03^d^	<0.001
n-6/n-3 PUFA	32.94 ± 9.21^a^	19.66 ± 4.15^b^	14.32 ± 4.03^c^	8.54 ± 1.57^d^	<0.001

ND means below the detection limit, not detected. Mean values marked with different letters in the same line indicate significant differences (*P* < 0.05). The other information is the same as Table 5.

### Correlation analysis

**FIGURE 1:**
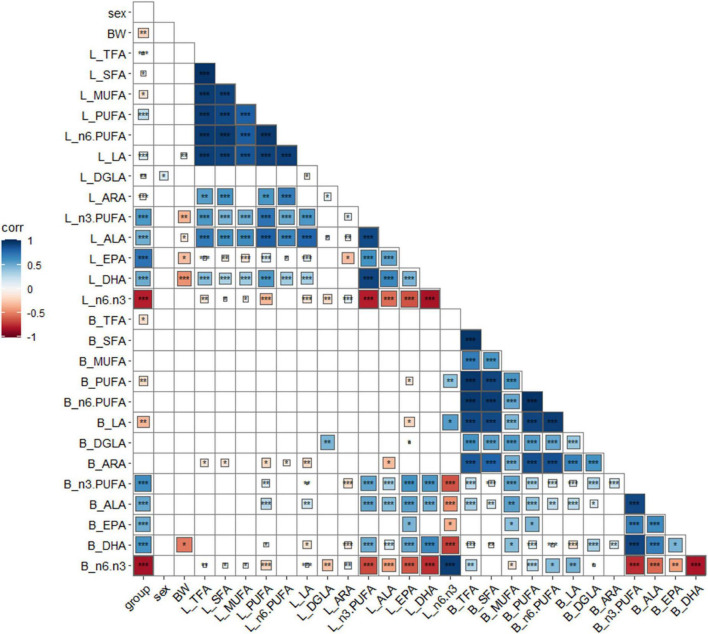
Correlation analysis of key indexes. *P < 0.05, **P < 0.01, ***P < 0.001, blue positive correlation, red negative correlation, darker color, higher correlation, lighter color, lower correlation. Group: different diet group; sex, sex; BW, body weight; B, breast muscle; L, liver.

Group, sex, BW, and FA composition of the liver and breast muscle were analyzed for correlation analysis, and the results are shown in Figure 1. Linseed oil supplementation was positively (*P* < 0.001) correlated with n-3 PUFA levels in the liver and breast muscle and negatively (*P* < 0.001) correlated with the proportion of n-6: n-3 PUFA. Sex was not correlated with most indexes (*P* > 0.05) but was positively (*P* < 0.05) correlated with liver DGLA levels. BW was negatively correlated (*P* < 0.05) with n-3 PUFA levels, especially DHA levels, in the liver (*P* < 0.001). Total fatty acids, saturated fatty acids, monounsaturated fatty acids, and n-6 PUFAs in the liver and breast muscle showed little correlation; however, only DGLA in the liver and breast muscle showed a positive correlation (*P* < 0.01). n-3 PUFA, ALA, EPA, and DHA in the liver were positively (*P* < 0.001) correlated, and other n-3 PUFAs in the breast muscle were also significantly positively correlated (*P* < 0.001), except for EPA (with was undetected). There was a significant positive (*P* < 0.001) correlation between n-3 PUFAs in the liver and breast muscle (except for breast muscle EPA). These results indicate that dietary n-3 PUFAs had a great influence on the fatty acid composition of the body, and there was a correlation between the n-3 PUFA composition of the liver and breast muscle.

### Overview of transcriptome data

**FIGURE 2:**
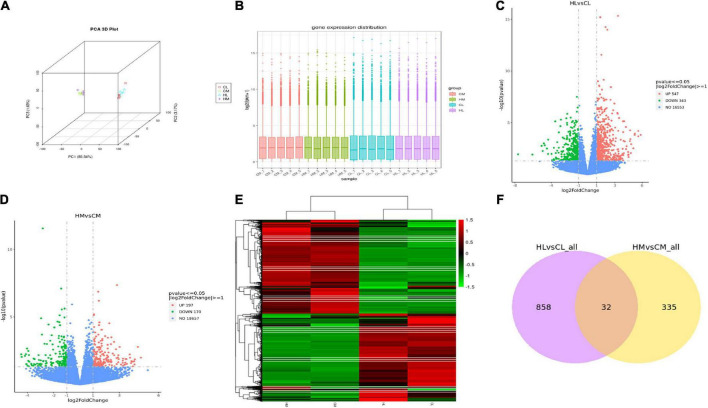
Transcriptomic analysis of liver and breast muscle tissues in the high linseed oil and control groups. **(A)** PCA 3D Plot of samples. **(B)** Box plot of sample gene expression distribution. **(C)** Volcano plots of the DEGs between HL and CL. **(D)** Volcano plots of the DEGs between HM and CM. **(E)** Cluster heatmap of the DEGs, red: upregulated genes; green: downregulated genes. **(F)** Venn diagram indicated unique and shared DEGs between HL vs. CL and HM vs. CM.

RNA-seq was performed on 10 male individuals: five duck livers or breast muscles with the lowest n-3 PUFA ratios (CL1, CL2, CL3, CL4, CL5 and CM1, CM2, CM3, CM4, CM5) and five duck livers with the highest n-3 PUFA ratios (HL1, HL2, HL3, HL4, HL5 and HM1, HM2, HM3, HM4, HM5). The PUFA compositions are shown in Supplementary Table 2. The read numbers and quality are shown in Supplementary Table 3. Clean reads were obtained after removing the low-quality sequences. The results indicated that more than 81% of the total clean reads of the 20 liver and breast muscle samples matched the reference sequences and more than 66% of the total clean reads were uniquely mapped to the reference sequences. The results demonstrated that all samples met the requirements for subsequent analysis. To assess intergroup differences and intragroup sample duplication, we performed PCA analysis on FPKM and read count of all samples, and the results showed same tissue aggregation and intergroup isolation (Figure 2A). The distribution of gene expression levels in different samples was shown in Figure 2B. And such data could produce a reliable basis for subsequent analysis.

### Identification of differentially expressed genes and functional enrichment analysis

A total of 9,859 genes were annotated in the duck livers and 10,426 genes were annotated in the duck breast muscles. With FDR < 0.05 and | log2-fold change| > 1 as the threshold, a total of 890 hepatic DEGs were identified including 547 upregulated genes and 343 downregulated genes (Figure 2C), and a total of 367 breast muscle DEGs were identified including 197 upregulated genes and 170 downregulated genes (Figure 2D). Other compassions were performed in Supplementary Figures 1A,B. There were 7876 DEGs between HL and HM, and there were 8003 DEGs between CL and CM. A hierarchical cluster analysis was performed by all DEGs, as shown in Figure 2E, there was considerable variation between livers and breast muscles. There were 32 common DEGs between HL vs. CL and HM vs. CM (Figure 2F). While there were 6785 common DEGs between HL vs. HM and CL vs. CM (Supplementary Figure 1C).

**FIGURE 3:**
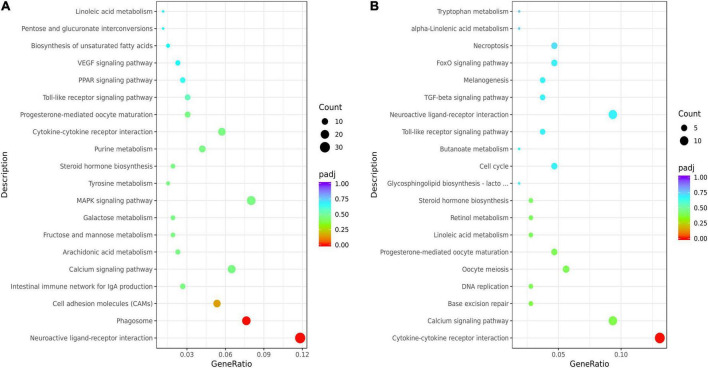
Kyoto Encyclopedia of Genes and Genomes (KEGG) enrichment analysis of the liver or breast muscle samples affected by linseed oil. **(A)** Bubble diagram of the top 20 KEGG pathway enrichments for the HL vs. CL comparisons. **(B)** Bubble diagram of the top 20 KEGG pathway enrichments for the HM vs. CM comparisons. The ordinate indicates the pathways.

The KEGG analysis showed that the top 20 KEGG terms were showed in Figure 3. And there were 7 common pathways (cytokine-cytokine receptor interaction, calcium signaling pathway, progesterone-mediated oocyte maturation, linoleic acid metabolism, steroid hormone biosynthesis, toll-like receptor signaling pathway, and neuroactive ligand-receptor interaction) enriched by DEGs from both HL vs. CL and HM vs. CM. Differential genes in livers were mainly enriched in biosynthesis of unsaturated fatty acids (downregulated *SCD*, *FADS1*, *FADS2*, upregulated *ACOT6*), PUFAs (LA, ARA) metabolism (upregulated *PTGS2*, *TBXAS1*, *PLA2G4A*, and *CYP3A40*, downregulated *GPX2, CYP2C16*), PPAR signaling pathway (upregulated *ANGPTL4*, *CPT1B*, *ACSL5*, downregulated *SCD*, *FABP2*), steroid hormone biosynthesis (upregulated *CYP3A40*, *ST2B1*, downregulated *COMT*, *CYP7A1*, and *CYP2C16*). Differential genes in breast muscles were mainly enriched in LA or ALA acid metabolism (upregulated *CYP3A1*, *PLA2*), cytokine-cytokine receptor interaction (upregulated *IL16*, *INHBA*, *CX3CL1*, *TNFRSF17*, *CCL20*, downregulated *CNTFR*, *BMPR1B*, *CXCL14*), calcium signaling pathway (upregulated *HRH1*, *MCOLN2*, *P2RX5*, *OXTR*, *MCOLN3*, *BDKRB1*, downregulated *CAMK1G*, *ADCY8*), and steroid hormone biosynthesis (upregulated *CYP3A1*, *ST2B1*, downregulated *CYPC21*). Of course, because of the difference in tissue function between the liver and breast muscles, there were 12 common pathways enriched by DEGs from both HL vs. HM and CL vs. CM included by biosynthesis of unsaturated fatty acids, PPAR signaling pathway, primary bile acid biosynthesis, cardiac muscle contraction, calcium signaling pathway (Supplementary Figure 2).

### RT-qPCR validation of key genes

**FIGURE 4:**
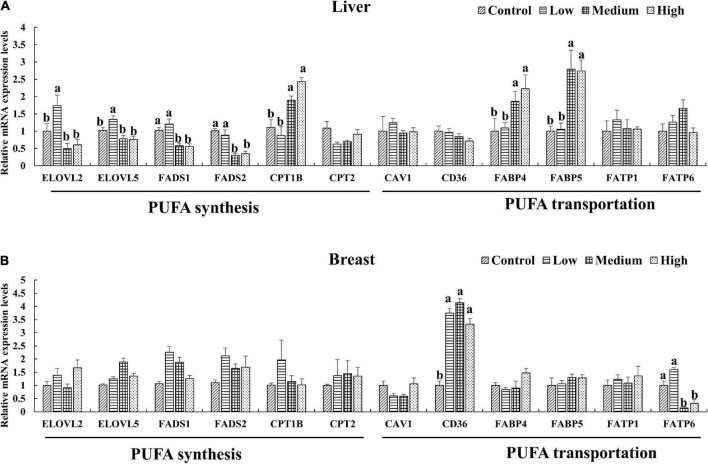
Expression of key genes verified by RT-qPCR in liver **(A)** and breast muscle **(B)**. Mean values marked with different letters in the same column indicate significant differences (*P* < 0.05, *n* = 8).

Since the PUFA metabolic pathways were involved by the DEGs, We further selected the key genes (*ELOVL2*, *ELOVL5*, *FADS1*, *FADS2*, *CPT1B*, *CPT2* and *CAV1*, *CD36*, *FABP4*, *FABP5*, *FATP1*, *FATP6*) related to n-3 polyunsaturated FA synthesis and transportation for quantitative verification. The expression levels of genes in the liver and breast muscle of ducks were shown in Figures 4A,B. The result was coincide with that of transcriptome result (Supplementary Figure 3). Dietary linseed oil supplementation had significant effects (*P* < 0.05) on the expression levels of liver desaturase and carbon chain extension enzyme genes. With the increase in linseed oil level, the expression levels of most genes first increased and then decreased. *ELOVL2* and *ELOVL5* mRNA expression levels were higher (*P* < 0.05) in the low linseed oil group than in the control, medium, and high linseed oil groups. Hepatic *FADS1* and *FADS2* mRNA expression levels were higher (*P* < 0.05) in the control and low-linseed-oil groups than in the medium and high-linseed-oil groups. Although there were changes in these genes in breast muscle, the differences were not statistically significant (*P* > 0.05). Hepatic *FABP4* and *FABP5* mRNA expression levels increased significantly (*P* < 0.05) in the medium and high linseed oil groups compared to that in the control and low linseed oil groups. Breast muscle *CD36* mRNA expression increased (*P* < 0.05) in all linseed oil groups, whereas the expression levels of *FATP6* were lower (*P* < 0.05) in the medium and high linseed oil groups than in the control and low linseed oil groups.

### Integrative analysis the effects of linseed oil on the polyunsaturated fatty acid transport and synthesis pathways in the liver and breast muscle

**FIGURE 5:**
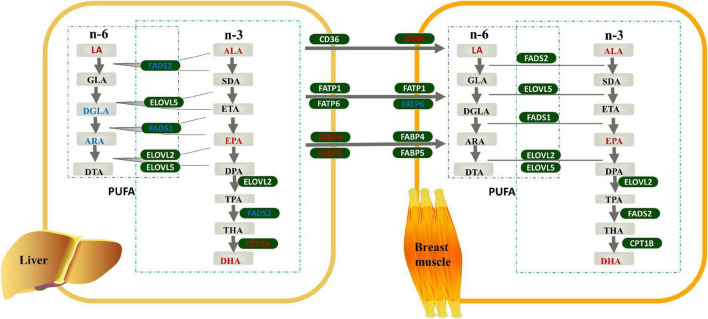
Effects of linseed oil on the polyunsaturated fatty acid (PUFA) synthesis and transport pathways in duck liver and breast muscle. Green boxes indicate genes, gray boxes indicate metabolites, red font indicate upregulated, blue font indicate downregulated.

Combined with the results of the FAs profile and genes expression analyses, we constructed a model diagram of the effect of linseed oil on the liver and breast muscle of ducks (Figure 5). Supplementation with linseed oil increased the synthesis of n-3 PUFA and the content of LA in the liver and breast muscle and decreased the synthesis of n-6 PUFA downstream of LA in the liver. Moreover, linseed oil had a significant influence on PUFA synthesis in the liver. Based on the shared enzyme system of n-6 and n-3 PUFA synthesis, the different distribution of enzymes caused by the substrate ALA may be the main reason for the change in the liver PUFA profile. *FABP4* and *FABP5*, which play an important role in liver PUFA transport, were upregulated in this organ. Linseed oil had little effect on PUFA synthesis genes in breast muscle, but the expression levels of *CD36* increased significantly with the increase in linseed oil, suggesting that PUFA in breast muscle may be partly derived from the transport of other tissues such as the liver, and PUFA transporters *CD36* play an important role in this process.

## Discussion

Linseed oil is the richest terrestrial source of n-3 fatty acids; it contains more than 35% ALA. n-3 PUFA have several health benefits, and high levels of such FAs in meat products would be desirable. Runzhou white-crested ducks were small sized meat ducks, with a marketing weight of about 1.4 kg. We evaluated the effects of dietary linseed oil on the liver and breast muscle of ducks. The results of this study reveal that diets with different amounts of linseed oil levels did not influence the body weight, ADFI, or FCR in ducks during the 28 d phase when dietary oil was supplemented at 0–2%. Similar findings have been reported in poultry fed diets supplemented with linseed oil (4, 20). This result could be attributed to the similar fat content in the experimental diets or the similar feed intake during the trial. Dietary linseed oil ratios had no effects on physical properties or chemical composition in duck breast muscle, which is consistent with corresponding results of both chickens and ducks (4, 21). While the study in pigs showed that high PUFA supplementation concentration improved IMF content and reduced meat color L* and pH 24 h, whereas low concentration decreased meat color b* and increased ADFI (22). Further, breast-meat sensory quality is not affected in broilers fed 100 g/kg flaxseed for 14 days (23). However, significant fishy odor and taste were recorded when chickens are fed a diet containing 7% linseed oil, whereas no significant differences in texture, tenderness, or juiciness of breast meat were reported (24).

In contrast to mammals, fat synthesis in birds is greater in the hepatic tissue and very limited in the adipose tissue. Fats that are metabolized in the liver are derived from three main sources: dietary fat, depot fat, and fat from *de novo* fatty acid synthesis (25). Dietary fatty acid composition and animal synthesis determine the fatty acid composition of the liver. Linseed oil is a valuable source of ALA in poultry diets and is effectively incorporated from the feed to the bird’s tissues (26). The n-3 PUFA synthesis pathway involves *FADS1*, *FADS2*, *ELOVL2*, and *ELOVL5*, which are affected by the nutritional status of the organism (11, 27, 28). Poultry have been shown to have strong elongating and desaturating activity during PUFA synthesis (10, 29, 30). Dietary linseed oil increases n-3 PUFA (ALA) concentrations and decreases n-6: n-3 PUFA ratios in birds fed diets. In this study, the contents of n-3 PUFAs (ALA, EPA, and DHA) increased in duck liver and breast muscle with increasing dietary linseed oil ratios, which is consistent with previous reports (31, 32). Correlation analysis results show that sex had no effect on most PUFA levels in breast muscles or livers, and previous studies have shown that sex had little effect on fatty acid composition in breast muscle and liver (33). In addition, most existing studies have focused on the detection of FA composition, and few studies have conducted correlation analysis. The results show that different linseed oil supplementation levels caused changes in fatty acids in the liver and breast muscle, and the content of n-3 fatty acids was significantly increased. There was no significant correlation between total fatty acids, SFA, MUFA, and n-6 PUFAs in the liver and breast muscle, whereas n-3 PUFA, ALA, EPA, and DHA levels in the liver and breast muscle were positively correlated, indicating that the change in n-3 PUFA induced by daily grain intake was systemic. The status of n-3 PUFA in other tissues can be roughly inferred from the level of n-3 PUFA in known tissues. In addition, BW was negatively correlated with n-3 PUFA levels, especially DHA levels, in the liver. Animal and human studies have also revealed that supplementation with EPA and DHA can prevent obesity and possibly improve BW in obese animals and humans (34).

Compared with the control group, the expression of genes related to polyunsaturated fatty acid synthesis, such as *SCD*, *FADS1*, and *FADS2*, were downregulated, while the expression of genes related to polyunsaturated fatty acid metabolism, such as *PTGS2*, *TBXAS1*, and *PLA2G4A*, was upregulated in the liver of ducks in the high linseed oil group. Cytosolic phospholipase A2 (cPLA2, PLA2G4A) catalyzes the release of arachidonic acid for prostaglandin synthesis by cyclooxygenase 1 (PTGS1), cyclooxygenase 2 (PTGS2), and thromboxane synthase (TBXAS1) (35). The DEGs, *PTGS2*, *TBXAS1*, and *PLA2G4A*, play important roles in downstream metabolites synthesis. n-6 PUFA (ARA) and n-3 PUFA (EPA, DHA) are two kinds of competitive substrates for the enzymes and products of PUFA metabolism (6). DHA- and EPA-derived eicosanoids antagonize the pro-inflammatory effects of ARA is as the substrate converted to prostaglandins, leukotrienes and other lipoxygenase or cyclooxygenase products. These products are important regulators of cellular functions with inflammatory, atherogenic and prothrombotic effects (2). Dietary linseed oil increased the synthesis of n-3 PUFAs in liver and promoted the expression of genes related to downstream anti-inflammatory metabolites synthesis, which is speculated to maintain the healthy state of ducks. Previous studies mainly focused on quantitative detection of genes related to PUFA synthesis, but little attention has been paid to the effect of dietary linseed oil on muscle transcriptome level. And we found that linseed oil mainly induced changes in the expression levels of genes in breast muscles, such as *CYP3A1*, *PLA2*, *ST2B1*, *IL16*, *INHBA*, *TNFRSF17*, which related to PUFA (linoleic acid or alpha-linolenic acid) and its metabolites (glycosphingolipid, steroid hormone, progesterone) metabolic pathways.

Genes related to n-3 PUFA synthesis and transportation were further verified by RT-qPCR. Most of the hepatic genes involved in PUFA synthesis showed the highest expression level in the low linseed oil group, and, as the proportion of linseed oil further increased, the gene expression levels decreased. This finding is consistent with that described by Li et al. (4). And the expression of FADS2 is proved to be changed over time according to the dietary n-6: n-3 ratio, supporting that metabolic enzymes of long-chain PUFA are regulated by dietary fatty acid composition (36). Besides, researchers have suggested that the consumption of an n-3 enriched diet stimulates mRNA expression of *FADS2* owing to the higher affinity of *FADS2* for n-3 than for n-6 (33, 37). Interestingly, the conversion of n-3 and n-6 fatty acids shares the same desaturase and elongase series. There is competition between the n-3 and n-6 fatty acid families; an excess of one leads to a significant reduction in the conversion of the other (24). We also found that hepatic LA increased, whereas the downstream n-6 PUFA (DGLA and ARA) decreased with increasing dietary linseed oil ratios. This may reflect the possibility that the increase in n-3 PUFA synthesis does not depend on the increase in related enzymes but on the increase in substrates (ALA) and the allotted enzyme proportion. The increased synthesis of EPA and DHA using ALA as a substrate competitively inhibits the transformation of LA to downstream DGLA and ARA.

The fatty acid binding protein (*FABP*) family comprises a series of small proteins that act as lipid uptake and intracellular transporters, including *FABP4* and *FABP5* (38). Both play an important role in lipid-related metabolic processes and are associated with liver fat, independent of obesity (39). *FABP4/5*-deficient mice do not exhibit any extension in their lifespan (40). In duck livers, linseed oil caused high expression levels of *FABP4* and *FABP5*, and the supplement level of linseed oil was negatively correlated with the body weight. These results indicate that the increased expression levels of *FABP4* and *FABP5* in linseed oil-supplemented duck liver is related to the transport of PUFA, they but do not lead to body weight gain. *CD36* is a high-affinity receptor that functions in muscle and adipose tissues uptake of long-chain fatty acids (41). Furthermore, the increasing CD36 protein of the muscle or the translocation of CD36 from an intracellular pool to the plasma membrane can promote LCFA transport (42). Our results show that *CD36* was upregulated by linseed oil in the duck breast muscles. This is consistent with the results of previous studies (43, 44). FATPs are a group of transport proteins that mediate the uptake of long-chain fatty acids (45). The study in both human and mouse showed that sustained skin barrier disruption gives rise to increases in FATP1 and –6 levels, as well as a robust increase in the levels of CD36 protein (46). We found that linseed oil decreased the expression level of *FATP6*; however, whether this affects barrier changes in ducks is unknown, and there are few relevant studies on *FATP6*. The metabolic mechanism of PUFA in the duck body induced by dietary linseed oil is currently unclear and needs to be further investigated.

## Conclusion

Dietary linseed oil supplementation for 28 days had little adverse effect on the performance and breast meat quality of ducks, but it enriched the meat with ALA, EPA, and DHA and decreased the n-6-to-n-3 ratio. Therefore, it is appropriate for health-conscious consumers. The levels of n-3 PUFA, especially DHA, in the liver were negatively correlated with the BW of ducks. The liver is an important tissue for PUFA synthesis. Members of the n-6 and n-3 FUFAs compete for the corresponding desaturase and elongase enzymes. The increase in ALA reduces the expression of *FADS1* and *FADS2*, increases the expression of *PTGS2*, *TBXAS1*, and *PLA2G4A* in the liver, and the substrate-dependent PUFA metabolic pattern promotes the synthesis and metabolic pathways of n-3 PUFA, while inhibiting the synthesis and metabolic pathways of n-6 PUFA. n-3 PUFA in breast muscle is not only synthesized by itself but also derived from transport from other tissues, such as the liver. The expression levels of PUFA transport-related genes, such as *FABP4* and *FABP5* in the liver, and *CD36* in the breast muscle are increased. This study provides nutrition- and gene-regulated perspectives for n-3 PUFA-enriched poultry production and reference for the effects of linseed oil intake on lipid metabolism in human. Further studies are needed to explore the effect of ALA on the relationship between cells (liver or breast muscle) and PUFA metabolism.

## Data availability statement

The datasets presented in this study can be found in online repositories. The names of the repository/repositories and accession number(s) can be found below: https://www.ncbi.nlm.nih.gov/geo/, GSE214632.

## Ethics statement

The animal study was reviewed and approved by the Institutional Animal Care and Use Committee of Yangzhou University.

## Author contributions

GBC and LW conceived and designed the study. XH and AZ collected the samples. BD, LW, and ZW performed the experiments and analyzed the data. LW wrote the manuscript. GHC and ZW contributed to revisions of the manuscript. All authors read and approved the manuscript.

## Footnotes

1
https://ftp.ncbi.nlm.nih.gov/genomes/all/GCF/015/476/345/GCF_015476345.1_ZJU1.0/GCF_015476345.1_ZJU1.0_genomic.fna.gz

2
http://www.genome.jp/kegg/
