# Supplementary material for: Dietary linseed oil affects the polyunsaturated fatty acid and transcriptome profiles in the livers and breast muscles of ducks
Source: Front Nutr. 2022 Oct 28;9:1030712. doi: 10.3389/fnut.2022.1030712 (PMC9650093; doi:10.3389/fnut.2022.1030712)
Supplement: Supplementary file 1 [file Data_Sheet_1.docx]

Supplementary Table 1. Sequences of primer pairs of mRNA

| Gene | Forward | Reverse | products(bp) |
| --- | --- | --- | --- |
| *ELOVL2* | CCAAGGTGCTGTGGTGGTAT | CGCAGGGTATCCAGTTCAGG | 162 |
| *ELOVL5* | ATGAACTGGGTGCCTTGTGG | CAGATACGGACGCATTGCTG | 117 |
| *FADS1* | TTCCGTGAACTCCGTGTAGC | CCTGGACAGTGCCTAGAAGC | 181 |
| *FADS2* | TGCAACATCGAGCAGTCCTT | TGTTGGAAACAGGTGGTGCT | 81 |
| *CPT1B* | GCTACGGCGTCTCCTACATC | GATGTTCCTCCCGAAACGCT | 107 |
| *CPT2* | TATCCGTCCTGCCTCTATTC | GAGATGTCGGTCAAATCCCT | 166 |
| *CAV1* | AGGGCAACATCTACAAGCCC | GTTCCCTCTGGCTCAGCAAT | 172 |
| *CD36* | TCGTTTCGCAGTTCCTCGTGAAG | AGCTGTTGTGCAGTTCTGGGATATG | 103 |
| *FABP4* | GTGTGGCCAAGCCCAATGTA | TAGTCCCTTTGCCATCCCAC | 207 |
| *FABP5* | GATGGCAGAAAAACTCAGACGC | TCCATCCACAAGTTTCCGTGT | 111 |
| *FATP1* | GCCGGATTTTGCCCAATGTT | CGCAGGGGATCCTGTTGATT | 154 |
| *FATP6* | ACCTTTGTAACCAGCCCAGG | GGGCCAAACCTCATCAGGAA | 130 |
| *β-ACTIN* | ATGTCGCCCTGGATTTCG | CACAGGACTCCATACCCAAGAA | 165 |
| *GAPDH* | ATGTTCGTGATGGGTGTGAA | CTGTCTTCTGTGTGGCTGT | 175 |

*ELOVL2*, elongation of very long-chain fatty acids enzyme 2; *ELOVL5*, elongation of very long-chain fatty acids enzyme 5; *FADS1*, fatty acid desaturase 1; *FADS2*, fatty acid desaturase 2; *CPT1B*, carnitine palmitoyltransferase 1B; *CPT2*, carnitine palmitoyltransferase 2; *CAV1*, Caveolin 1; *CD36*, cluster of differentiation 36; *FABP4*, fatty acid binding protein 4; *FABP5*, fatty acid binding protein 5; *FATP1*, fatty acid transport protein 1; *FATP6*, fatty acid transport protein 6; *β-ACTIN*, Beta-actin; *GAPDH*, glyceraldehyde-3-phosphate dehydrogenase.

Supplementary Table 2. Fatty acid composition of liver samples used for RNA-seq

| Sample | TFA | n-6 PUFA | n-3 PUFA | ALA(C18:3n-3) | EPA(C20:5n-3) | DHA(C22:6n-3) | n-6/n-3 |
| --- | --- | --- | --- | --- | --- | --- | --- |
| CL.1 | 2.29 | 90.50 | 6.26 | 0.62 | 0.35 | 5.29 | 14.46 |
| CL.2 | 2.03 | 80.46 | 4.66 | 0.55 | 0.73 | 3.38 | 17.27 |
| CL.3 | 2.36 | 93.48 | 8.68 | 1.28 | 0.50 | 6.90 | 10.77 |
| CL.4 | 2.46 | 92.02 | 6.05 | 0.64 | 0.33 | 5.08 | 15.21 |
| CL.5 | 2.34 | 91.30 | 7.13 | 0.57 | 0.33 | 6.23 | 12.81 |
| **Average** | **2.30** | **89.55** | **6.56** | **0.73** | **0.45** | **5.38** | **14.10** |
| HL.1 | 2.70 | 81.93 | 32.76 | 14.10 | 3.46 | 15.20 | 2.50 |
| HL.2 | 3.78 | 127.85 | 30.14 | 11.67 | 2.83 | 15.64 | 4.24 |
| HL.3 | 2.73 | 99.03 | 28.41 | 8.81 | 1.74 | 17.86 | 3.49 |
| HL.4 | 2.65 | 81.84 | 23.42 | 15.27 | 1.13 | 7.02 | 3.49 |
| HL.5 | 1.98 | 72.73 | 20.81 | 4.48 | 2.05 | 14.28 | 3.49 |
| **Average** | **2.77** | **92.68** | **27.11** | **10.87** | **2.24** | **14.00** | **3.44** |
| CM.1 | 84.65 | 32.29 | 1.13 | 0.20 | 0.10 | 0.83 | 28.58 |
| CM.2 | 58.14 | 23.44 | 0.83 | 0.20 | 0.10 | 0.52 | 28.40 |
| CM.3 | 70.44 | 30.82 | 1.11 | 0.11 | 0.08 | 0.91 | 27.88 |
| CM.4 | 74.18 | 31.11 | 1.26 | 0.17 | 0.10 | 0.99 | 24.72 |
| CM.5 | 56.92 | 22.81 | 0.85 | 0.17 | 0.06 | 0.62 | 26.86 |
| **Average** | **68.87** | **28.09** | **1.03** | **0.17** | **0.09** | **0.77** | **27.29** |
| HM.1 | 62.37 | 24.32 | 4.29 | 0.96 | 0.59 | 2.74 | 5.67 |
| HM.2 | 95.70 | 37.22 | 4.55 | 1.01 | 0.61 | 2.92 | 8.18 |
| HM.3 | 66.39 | 27.55 | 3.07 | 0.63 | 0.49 | 1.95 | 8.97 |
| HM.4 | 94.79 | 39.79 | 4.30 | 1.02 | 0.85 | 2.43 | 9.25 |
| HM.5 | 64.61 | 28.00 | 3.92 | 0.63 | 0.51 | 2.78 | 7.14 |
| **Average** | **76.77** | **31.38** | **4.03** | **0.85** | **0.61** | **2.57** | **7.84** |

Note: CL means duck liver of control group, HL means duck liver of high linseed oil group, TFA for CL means total fatty acid (g/100 g), TFA for HM and other fatty acid components (mg/100 g).

Supplementary Table 3. Summary of genome mapping of mRNA libraries

| sample | total_reads | total_map | unique_map | multi_map | positive_map | negative_map |
| --- | --- | --- | --- | --- | --- | --- |
| CL.1 | 38856160 | 36532865(94.02%) | 34153563(87.9%) | 2379302(6.12%) | 17058601(43.9%) | 17094962(44.0%) |
| CL.2 | 43041066 | 38926912(90.44%) | 36463132(84.72%) | 2463780(5.72%) | 18199270(42.28%) | 18263862(42.43%) |
| CL.3 | 43061584 | 38840115(90.2%) | 35362336(82.12%) | 3477779(8.08%) | 17675870(41.05%) | 17686466(41.07%) |
| CL.4 | 44642404 | 40317386(90.31%) | 35738257(80.05%) | 4579129(10.26%) | 17821590(39.92%) | 17916667(40.13%) |
| CL.5 | 43512064 | 39278534(90.27%) | 36324972(83.48%) | 2953562(6.79%) | 18144114(41.7%) | 18180858(41.78%) |
| HL.1 | 42685086 | 38609807(90.45%) | 35065813(82.15%) | 3543994(8.3%) | 17565431(41.15%) | 17500382(41.0%) |
| HL.2 | 42221980 | 38501811(91.19%) | 34722263(82.24%) | 3779548(8.95%) | 17353271(41.1%) | 17368992(41.14%) |
| HL.3 | 43540884 | 39983335(91.83%) | 36922367(84.8%) | 3060968(7.03%) | 18436951(42.34%) | 18485416(42.46%) |
| HL.4 | 43457360 | 39066213(89.9%) | 35792605(82.36%) | 3273608(7.53%) | 17863033(41.1%) | 17929572(41.26%) |
| HL.5 | 41608410 | 38346754(92.16%) | 35928053(86.35%) | 2418701(5.81%) | 17928274(43.09%) | 17999779(43.26%) |
| CM.1 | 43314154 | 36324151(83.86%) | 29154756(67.31%) | 7169395(16.55%) | 14581633(33.66%) | 14573123(33.65%) |
| CM.2 | 43922074 | 36985279(84.21%) | 30197160(68.75%) | 6788119(15.45%) | 15063172(34.3%) | 15133988(34.46%) |
| CM.3 | 41914364 | 34805619(83.04%) | 28640659(68.33%) | 6164960(14.71%) | 14328676(34.19%) | 14311983(34.15%) |
| CM.4 | 45664960 | 38489038(84.29%) | 30867240(67.6%) | 7621798(16.69%) | 15410245(33.75%) | 15456995(33.85%) |
| CM.5 | 40744518 | 34344284(84.29%) | 29098926(71.42%) | 5245358(12.87%) | 14545047(35.7%) | 14553879(35.72%) |
| HM.1 | 44198082 | 37029208(83.78%) | 30545034(69.11%) | 6484174(14.67%) | 15293969(34.6%) | 15251065(34.51%) |
| HM.2 | 44651116 | 36571859(81.91%) | 30143744(67.51%) | 6428115(14.4%) | 15102788(33.82%) | 15040956(33.69%) |
| HM.3 | 40382666 | 34254770(84.83%) | 28741096(71.17%) | 5513674(13.65%) | 14394790(35.65%) | 14346306(35.53%) |
| HM.4 | 43538364 | 37479310(86.08%) | 29061443(66.75%) | 8417867(19.33%) | 14531725(33.38%) | 14529718(33.37%) |
| HM.5 | 41524340 | 35689983(85.95%) | 29845758(71.88%) | 5844225(14.07%) | 14944904(35.99%) | 14900854(35.88%) |

Note: sample; total_reads; clean readsl; total_map: number and percentage of reads compared to the genome; unique_map: the number and percentage of reads compared to a unique location in the reference genome (for subsequent quantitative data analysis of reads); multi_map: number and percentage of reads compared to multiple locations in the reference genome; positive_map: the number and percentage of reads compared to the positive strand of the reference genome; negative_map: the number and percentage of reads compared to the negative strand of the reference genome.
