# Supplementary figures and images for: Dietary linseed oil affects the polyunsaturated fatty acid and transcriptome profiles in the livers and breast muscles of ducks
Source: Front Nutr. 2022 Oct 28;9:1030712. doi: 10.3389/fnut.2022.1030712 (PMC9650093; doi:10.3389/fnut.2022.1030712)

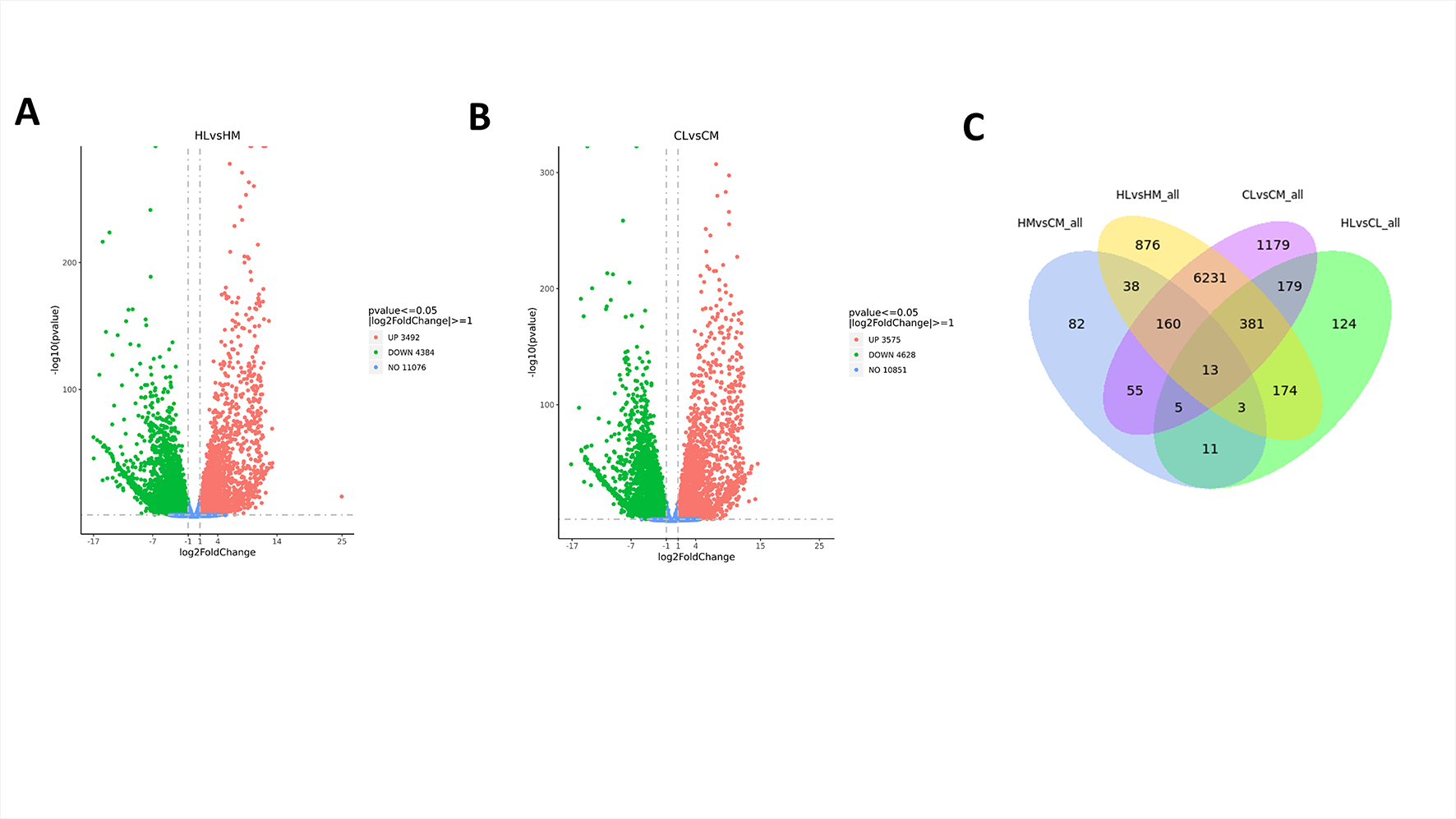

Supplement: Supplementary file 2 [file Image_1.TIF]

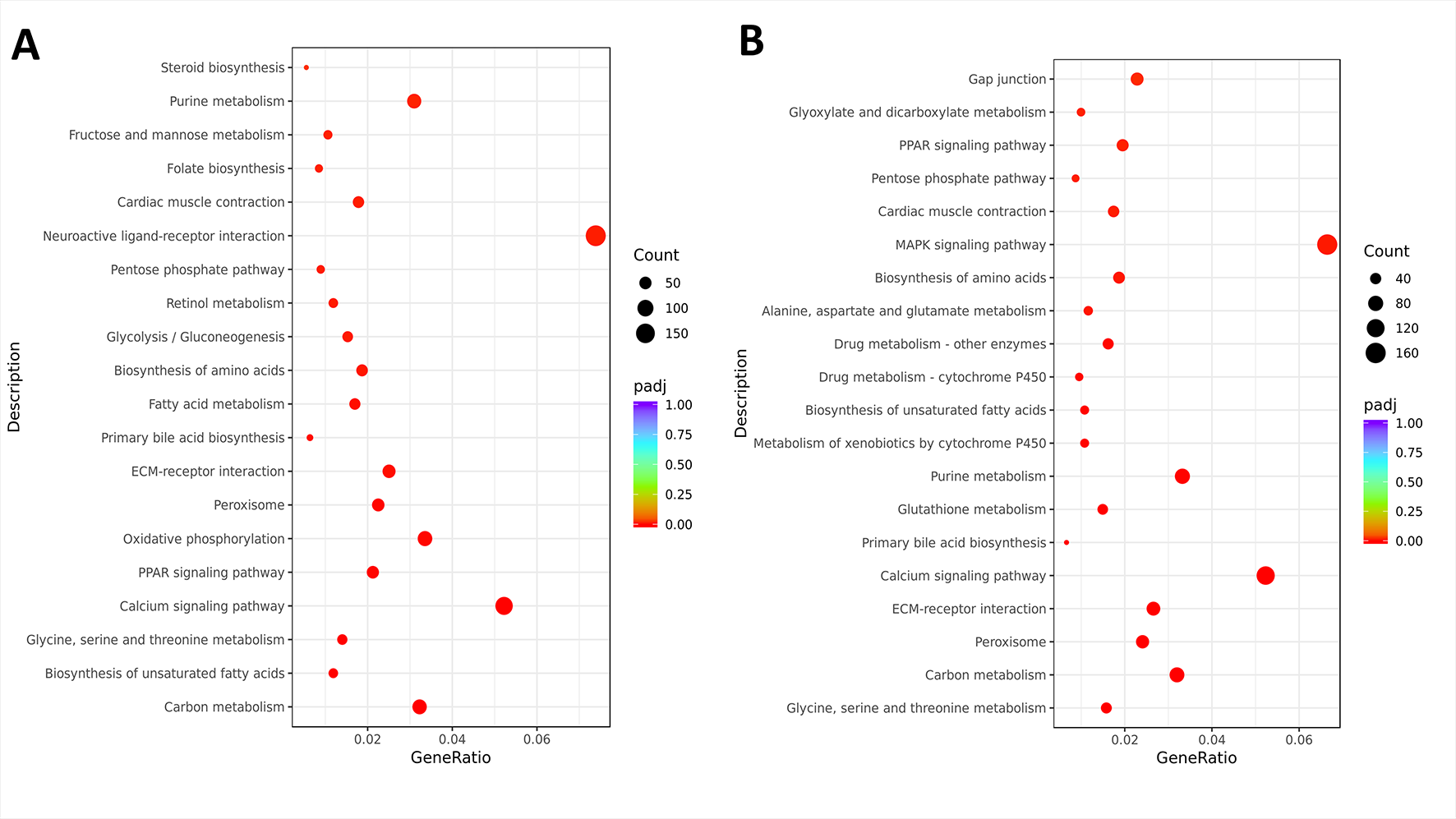

Supplement: Supplementary file 3 [file Image_2.TIF]

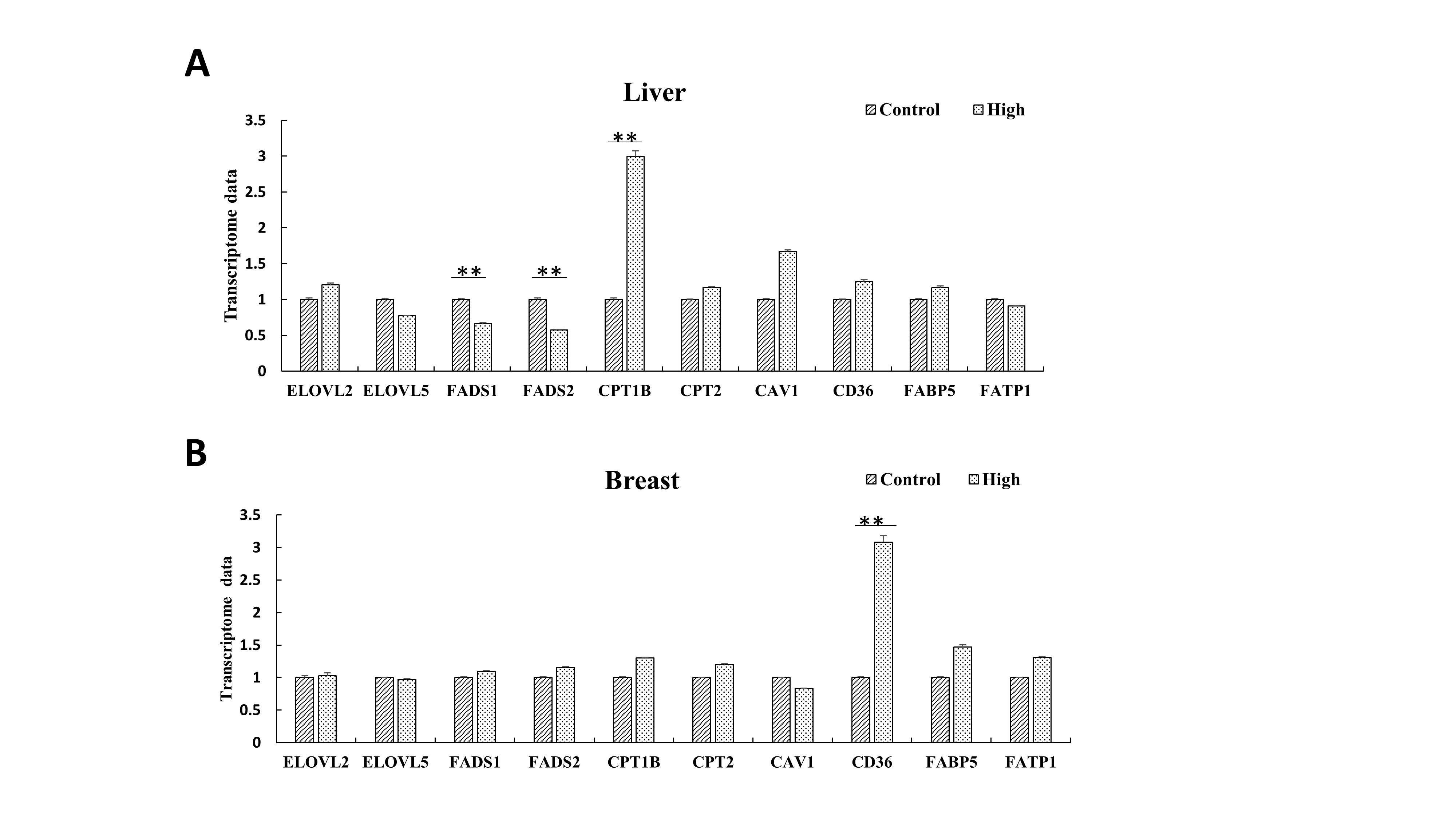

Supplement: Supplementary file 4 [file Image_3.PNG]
